# Socio-spatial cognition in cats: Mentally mapping owner’s location from voice

**DOI:** 10.1371/journal.pone.0257611

**Published:** 2021-11-10

**Authors:** Saho Takagi, Hitomi Chijiiwa, Minori Arahori, Atsuko Saito, Kazuo Fujita, Hika Kuroshima

**Affiliations:** 1 Department of Psychology, Graduate School of Letters, Kyoto University, Sakyo, Kyoto, Japan; 2 Japan Society for the Promotion of Science, Chiyoda-ku, Tokyo, Japan; 3 Research and Development Section, Anicom Speciality Medical Institute Inc., Yokohamashi-Nakaku, Kanagawaken, Japan; 4 Wildlife Research Center, Kyoto University, Sakyo, Kyoto, Japan; 5 Department of Psychology, Faculty of Human Sciences, Sophia University, Chiyoda-ku, Tokyo, Japan; Texas Christian University, UNITED STATES

## Abstract

Many animals probably hold mental representations about the whereabouts of others; this is a form of socio-spatial cognition. We tested whether cats mentally map the spatial position of their owner or a familiar cat to the source of the owner’s or familiar cat’s vocalization. In Experiment 1, we placed one speaker outside a familiar room (speaker 1) and another (speaker 2) inside the room, as far as possible from speaker 1, then we left the subject alone in the room. In the habituation phase, the cat heard its owner’s voice calling its name five times from speaker 1. In the test phase, shortly after the 5^th^ habituation phase vocalization, one of the two speakers played either the owner’s voice or a stranger’s voice calling the cat’s name once. There were four test combinations of speaker location and sound: Same_sound_Same_location_, Same_sound_Diff_location_, Diff_sound_Same_location_, Diff_sound_Diff_location_. In line with our prediction, cats showed most surprise in the Same_sound_Diff_location_ condition, where the owner suddenly seemed to be in a new place. This reaction disappeared when we used cat vocalizations (Experiment 2) or non-vocal sounds (Experiment 3) as the auditory stimuli. Our results suggest that cats have mental representations about their out-of-sight owner linked to hearing the owner’s voice, indicating a previously unidentified socio-spatial cognitive ability.

## Introduction

Mental representations about the whereabouts of living things such as other group members, predators, or prey are likely to be advantageous for many animals, especially in conditions of poor visibility [1, 2]. Noë & Laporte called this ability “socio-spatial cognition” [1]; it can be seen as a valuable cognitive ability that involves spatially mapping others by using auditory information such as vocalizations or other auditory cues.

Mentally representing non-visible living things might be strongly related to “object permanence,” which involves maintaining a mental representation of an object even though it can no longer be directly perceived [3]. In humans, the ability is known to be present at a relatively early developmental stage [4]. Many non-human species have tested positively for at least some levels of object permanence (see review in [5]; for example, chimpanzees (*Pan troglodytes*): [6–9], bonobos (*Pan paniscus*): [6, 7], orangutans (*Pongo pygmaeus*): [6, 8], gorillas (*Gorilla gorilla*): [6, 10], Eurasian jays (*Garrulus glandarius*): [11], bears (*Melursus ursinus* and *Helarctos malayanus euryspilus*): [12], dogs (*Canis lupus familiaris*):[13] and cats (*Felis catus*): [14]). Subjects belonging to these diverse species searched in the correct place when food or another object was seen to disappear (visible displacement). Some apes even passed “invisible displacement” trials that required tracking of invisible object movements [15]. Almost all object permanence tests present the subjects with visual information only. But for animals in their natural environment, it could be important to mentally track other individuals’ whereabouts using other sensory modalities (e.g., audition).

Vervet monkeys (*Chlorocebus pygerythrus*) were shown to mentally track out-of-sight conspecifics through playbacks of their vocalizations [1]. The monkeys responded more when a group member appeared to be implausibly displaced, suggesting the ability to mentally follow invisible displacements of familiar individuals. Similar results were obtained in meerkats (*Suricata suricatta*) [16] and tropical birds: green woodhoopoes (*Phoeniculus purpureus*) [17]. Meerkats also showed strong responses when a conspecific suddenly appeared to be “teleported,” with its vocalization coming from a location other that the one where they heard the conspecific’s vocalization just a moment ago. Green woodhoopoes responded more rapidly to strangers and to neighbors in the wrong location than to neighbors in their expected location. In sum, animals that live in social groups or that hunt prey under conditions of poor visibility might be expected to hold mental representations of other animate things.

In this study, we tested cats (*Felis catus*). This animal has remarkable hearing ability and can use sounds to infer the location of unseen prey under conditions of low visibility [18]. Cats have sensitive ears that have more than 20 muscles and are capable of moving independently in all directions [19]. The auditory range for cats extends from 55 Hz to 79 kHz [20]. These ecological and anatomical features might promote cognitive processing of auditory information. Indeed, cats appear to be good at inferring physical and social presence from sounds. Takagi et al demonstrated this kind of inference for unseen inanimate objects [21], and Takagi et al showed that cats expected to see their owner’s face upon hearing their owner’s voice [22]. These studies provide evidence that cats can predict visual events based on auditory information.

Other studies have also shown that cats acquire social information from audition. They discriminate between their owner’s voice and a stranger’s [23], and they can recognize emotional sounds from other cats and humans, by matching vocalizations to facial expressions (Angry vs. Happy) [24]. In addition, they pass visible displacement tasks [25], confirming cats’ ability to form mental representations of objects. In sum, cats use sounds to identify individual humans and discriminate emotional states of humans and cats. Considering their natural crepuscular profile and hearing-dominant ecology along with their social cognitive abilities described above, it seems plausible that cats should be able to mentally map others’ locations based on vocalizations.

Here we tested whether cats hold mental representations of others and spatially map others based on auditory information in the form of vocalizations. To this aim, we presented cats with “teleportation”-like scenarios as used in studies with vervet monkeys [1] and meerkats [16]. In Experiment 1, the owner’s voice was played back sequentially from two separate locations, in a simulation of apparent teleportation. In Experiment 2 we presented a vocalization from a familiar cat to examine whether similar socio-spatial cognitive processing would occur with a conspecific vocalization. We used nonsocial physical sounds in Experiment 3 as a control condition, to test whether reactions seen in Experiments 1 and 2 were specific to social stimuli. As live agents move spontaneously, it is impossible for the same live agent to exist in two places at the same time, whereas it might be at least theoretically possible for non-social objects to replicate and occupy two locations simultaneously. For this reason, we included a non-social sound as a control condition. Our hypothesis was that if cats identify a familiar individual (owner or cat) from vocalizations and mentally map their location, they should react more strongly when the same sound is sequentially played back from different locations, simulating an “impossible teleportation” of the owner or familiar cat.

## General procedure

**Fig 1:**
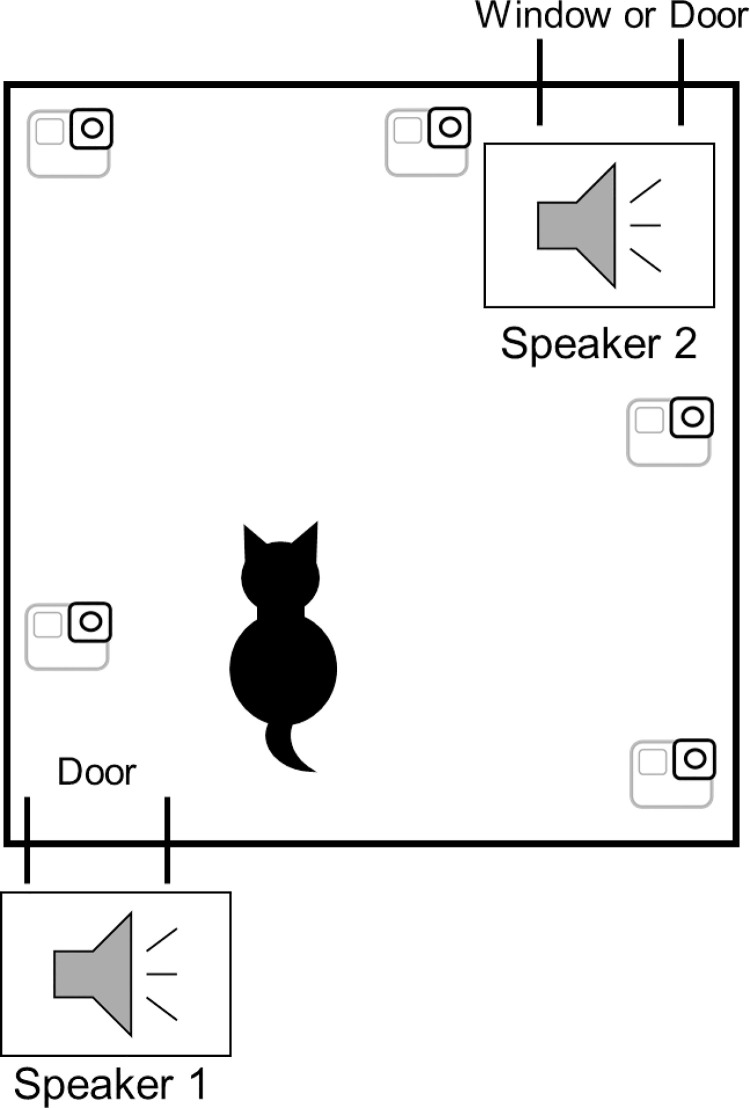
Arrangement of a testing room. There were slight differences across testing rooms depending on cats’ familiar spaces (house or cat café). The Experimenter placed speaker 1 outside the test room and speaker 2 inside the test room close to another door or window leading to another room or outside. Cats were left alone and could move freely. Cat behaviors were recorded by video cameras during tests.

The cats were individually tested in their familiar environment: house or café. In the latter case we tested cats in the café, each of which had at least two separate rooms, one of which served as the test room and the other as a waiting room. The owner and the experimenter were in the room next to the test room. We used two wireless speakers (SONY SRS-X11, Japan) connected by Bluetooth. Speaker 1 was placed outside the test room, near the door. Speaker 2 was placed inside the test room close to another door or a window (Fig 1). The distance between the two speakers varied depending on the room layout, but it was at least 4 m, large enough to make it impossible for anyone to move from one to the other in 2.5 sec. After the experimenter positioned the speakers and five cameras (SONY HDR-CX675, Japan; three GOPRO HERO 5, United states; RICOH Theta V, Japan) to focus on the cat in the room, the experimenter left the room, leaving the cat to move freely. After 3 min to allow the cat to acclimate and relax, the experimenter started a trial by pressing a key on the computer that controlled the presentation of the stimuli. Note that we determined that a cat was relaxed if it explored the room normally, without hiding.

Three experiments (2 tests and 1 control experiment) were conducted, with two types of sounds played in each experiment for each condition (Table 1). The sounds were different in each experiment, each of which consisted of two phases: habituation and test. In the habituation phase, a sound (habituation stimulus) was played five times from speaker 1, and then in the test phase either the same sound or a different sound (test stimulus) was played once from either speaker 1 or 2, for a total of six presentations during the trial. The test phase continued for 15 sec after the test stimulus was played, and during this time the cat’s behavior was video recorded. There were four conditions depending on “what” test stimulus was played back from “where”: Same_sound_Same_location_, Diff_sound_Same_location_, Same_sound_Diff_location_, and Diff_sound_Diff_location_. For example, in Diff_sound_Diff_location_, the sound used in the habituation phase was different from that used in the test phase, and the latter was played from speaker 2, which was also different from the one in the habituation phase (Table 1). Combinations of sounds (same or different) were between-subject conditions, while location of speaker (same or different) was a within-subject condition. Each cat participated once in each of two conditions, for example, Diff_sound_ Same_location_ and Diff_sound_Diff_location_. The order of conditions was counterbalanced across subjects.

**Table 1:** Explanation of conditions in Exp.1 to Exp.3.

	Habituation phase		Test phase	
	sounds	Location	sound	Location
Exp.1	Owner voices	1	Owner or Stranger voice	1 or 2
Exp.2	Cat A vocalizations	1	Cat A or Cat B vocalization	1 or 2
Exp.3	Physical sound A or B	1	Physical sound A or B	1 or 2

In Exp.1, we used owner voices as habituation sounds and owner or stranger voices as test sounds. In Exp.2 and Exp.3, we used familiar cats’ vocalization or physical sounds as habituation sounds and test sound.

The inter-stimulus interval (ISI) was 2.5 sec, and the inter-trial interval (ITI) was at least 3 min. During the ITI, cats could move freely in the room. All sounds were adjusted to be around 1 sec in duration, and volume was equated using Audacity® version 2.3.0 [26]. Presentations of sound stimuli were controlled via the Visual Studio 2013 program on a personal laptop computer (NEC Lavie G type Z, Japan).

We predicted that cats would be especially surprised by test stimuli in the Same_sound_Diff_location_ condition, in which the familiar individual was apparently teleported (Experiments 1 and 2), but that they would show no such response when non-social sounds were used (Experiment 3).

This study adhered to the ethical guidelines of Kyoto University, and was approved by the Animal Experiments Committee of the Graduate School of Letters, Kyoto University (No. 17–35). Rating procedures adhered to the ethical guidelines of Kyoto University and Bukkyo University. This study was approved by the unit for advanced study of mind, Kyoto University (30-P-18) and Human Experiment Committee, Bukkyo University (H30-27-A). We obtained informed consent from all owners both verbally and on paper. Cats were not deprived of water or food for any reason related to the study.

## Experiment 1

### Methods

#### Ethical statement

This study adhered to the ethical guidelines of Kyoto University, and was approved by the Animal Experiments Committee of the Graduate School of Letters, Kyoto University (No. 17–35). Rating procedures adhered to the ethical guidelines of Kyoto University and Bukkyo University. This study was approved by the unit for advanced study of mind, Kyoto University (30-P-18) and Human Experiment Committee, Bukkyo University (H30-27-A). We obtained informed consent from all owners both verbally and on paper. Cats were not deprived of water or food for any reason related to the study.

#### Subjects

Fifty domestic cats (*Felis catus*) (23 males, 27 females) participated in Exp.1. Twenty-seven were kept at five “cat cafés” (11 males, 16 females, mean age 4.81 years, *SD* = 3.36 years, range 11 months to 12.6 years), where visitors enjoy contact with the resident cats. Cafés cats lived in rooms with other cats. The remaining subjects were house cats (12 males, 11 females, mean age 6.61 years, *SD* = 4.02 years, range 11 months to 15.6 years) (see details in S1 Table). An additional five cats were excluded due to two of them staying out of camera range, and inadequate sound due to a poor Bluetooth connection (three cats).

#### Apparatus & stimuli

We used the owner’s voice calling the cat’s name as the habituation stimulus and/or test stimulus, and the voice of a same-sex unfamiliar person calling the cat’s name as a test stimulus. In cat cafés, we regarded the main caregivers as owners. We asked the owner to call the cat’s name as usual and recorded the call using a handheld digital audio recorder (SONY ICD-UX560F, Japan) in WAV format. The sampling rate was 44,100 Hz and the sampling resolution was 16-bit. The call lasted about 1 sec, depending on the length of cat’s name (mean duration = 1.24 sec, *SD* = 0.21, average frequency = 222.92 Hz, *SD* = 114.87, mean loudness = -15.07 dB, *SD* = 2.12). Note that the habituation stimulus was always the owner’s voice, and the test stimulus was either owner’s voice or stranger’s voice in Exp.1 (see Table 1).

#### Analysis

We extracted a video clip for evaluation from 2 sec before the test stimulus sound onset until 12 sec after the sound offset for each test phase. Test sounds were replaced by pure tones for the purpose of blind coding of the clips. All data from “error” trials (nine due to a bad Bluetooth connection, two due to experimenter error, and seven due to the cat being out of camera range) were excluded from the analysis. In total, 82 clips were created using Adobe Premiere CC 2019 (USA): 20 for Same_sound_Same_location_, 22 for Diff_sound_Same_location_, 21 for Same_sound_Diff_location_, and 19 for Diff_sound_Diff_location_. We used subjective ratings by raters instead of coding some behaviors following Kubinyi, Gosling, and Miklósi’s report that such ratings are appropriate when the target concept consists of multiple behaviors [27]. Our target concept, “surprise”, included multiple behaviors including moving ears, head direction, looking back and displace. Therefore, we used subjective ratings in addition to latency to look back. Eight “blind” raters (4 men, 4 women, mean age 21 years) evaluated the subjects’ reaction in all clips, in random order. They were instructed to subjectively rate the cat’s level of surprise to the pure tones on a 5-point scale ranging from 0 (no surprised) to 4 (strongly surprised) (see video clips which show typical cats’ reaction rated 0 and 4 in supporting information). They were also instructed to ignore any reaction to other environmental sounds.

All statistical analyses were conducted using R version 3.5.1 [28]. The magnitude of surprise was analyzed by a linear mixed model (LMM) using a lmer function in lme4 package version 1.1.10 [29], in which location (same/diff), sound (same/diff), the interaction between location and voice type and trial order (first/second) were entered as fixed factors, and subject identity was entered as a random factor. To test whether factor effects were significant, we ran Wald chi square tests by an Anova function in car package [30]. We used a difflsmeans function in lmer Test package [31] which tested differences of least squares to compare each condition. Degrees of freedom were adjusted by Kenward-Roger, and p-value was adjusted using the Tukey method.

In addition to the ratings analysis, we analyzed latency to look toward speaker 2. One of the experimenters (H.C.), blind to the conditions, counted the number of frames (30 frames/sec.) until the subjects looked toward speaker 2 in all conditions. Looking onset was defined as the cat’s head starting to orient toward speaker 2. Although cats had no reason to look back in the Same_location_ conditions, we analyzed looking back as a control. Seventeen, 13, 13 and 8 cats who looked back toward speaker 2 during test phase for Same_sound_Diff_locaation_, Diff_sound_Diff_location_, Diff_sound_Same_location_ and Same_sound_Same_location_ conditions were included. The videos were analyzed using Adobe Premiere CC 2019. To check reliability of coding, an assistant who was blind to the conditions coded a randomly chosen 20% of the videos. The correlation between the two coders was high and positive (Pearson’s *r* = 0.92, *n* = 24, *p* < 0.01).

To analyze latency to look back at speaker 2, we ran a linear model (GLM) in which sound (same/diff), location (same/diff) and interaction were entered as fixed factors using a glmer function with Gamma distribution in lme4 package version 1.1.10 [29].

Finally, we computed correlations between magnitude of surprise and latency to look back at speaker 2, using Pearson’s product-moment correlation test to confirm whether raters judged “surprise” based on looking back.

### Results & discussion

#### Magnitude of surprise

**Fig 2:**
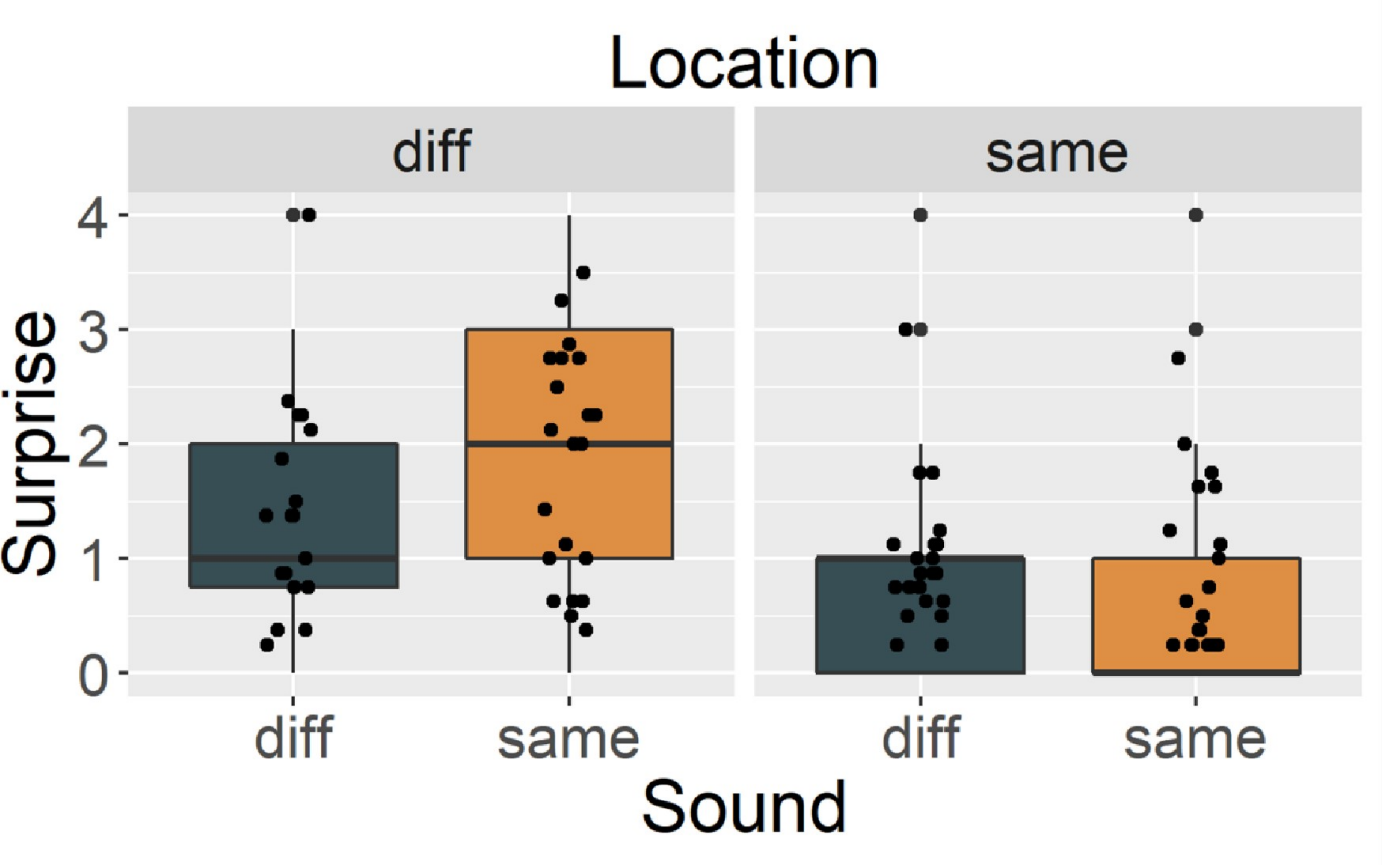
The magnitude of subjects’ surprise rated by eight blind raters in Exp.1. Black blue bar represents Diff_sound_. Orange bar represents Same_sound_. Left panel represents Diff_location_ and right panel represents Same_location_. Each point represents mean magnitude of surprise for each trial.

Fig 2 shows the magnitude of the cats’ surprise reactions. As predicted, the cats showed more surprise in Same_sound_Diff_location_ than the other conditions. LMM revealed an interaction of location and sound (*X*^2^ (1) = 5.675, *p* = .017). There was no significant main effect of trial order (*X*^2^ (1) = 1.763, *p* = .184) or sound (*X*^2^ (1) = 2.772, *p* = .095), which shows that cats did not react strongly when they heard a different voice. Multiple comparisons showed a significant difference between Same_sound_Diff_location_ and Diff_sound_Diff_location_ (*t*(628) = 2.871, *p* = .021), and between Same_sound_Diff_location_ and Same_sound_Same_location_ (*t*(628) = 7.357, *p* < .001). These results indicate that cats were most surprised in the Same_sound_Diff_location_ condition, possibly because they mentally mapped the owner’s location when they heard the owner’s voice in the habituation phase and were surprised by their “impossible teleportation” in the test phase. Note that we did not conduct a stranger habituation condition; the results might therefore be limited to when the owner’s voice is involved.

#### Latency to look at speaker 2

**Fig 3:**
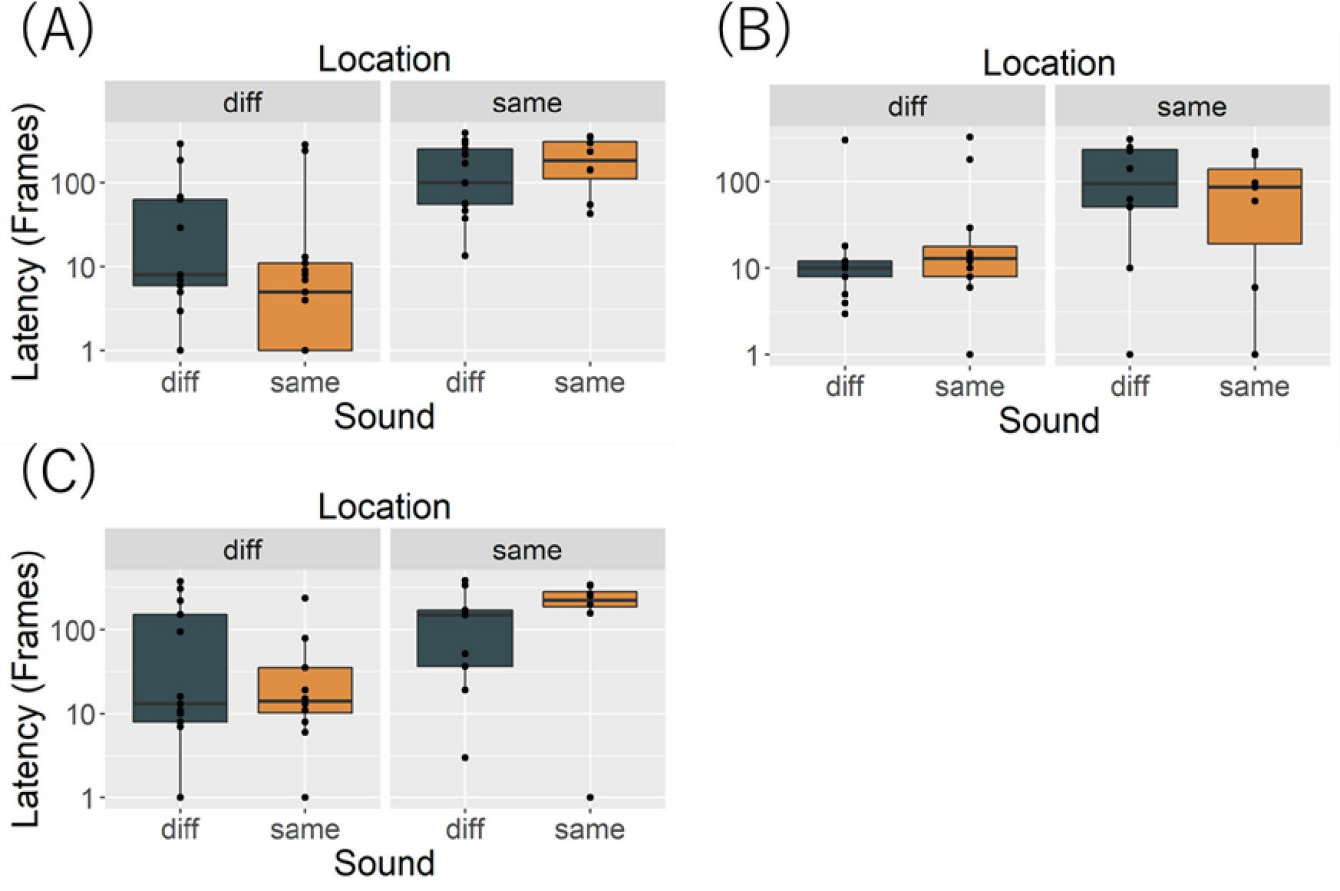
Latency to look back at speaker 2 in (A) Exp.1, (B) Exp.2, and (C) Exp.3. Orange boxplot represents Same_sound_, black blue boxplot represents Diff_sound_.

Fig 3(A) shows latencies to look toward speaker 2. Although cats appeared to look sooner in Same_sound_Diff_location_ condition, GLM revealed a significant main effect of location (*X*^2^ (1) = 8.303, *p* = .003), but not sound (*X*^2^ (1) = 0.019, *p* = .890) nor an interaction (*X*^2^ (1) = 0.457, *p* = .498). Looking toward speaker 2 did not vary as a function of the conditions, possibly because cats oriented regardless of the nature of the sound they heard.

#### Correlation between response magnitude and latency

Fig 6(A) shows the relationship between the magnitude of the cats’ surprise responses as rated by humans, and latency to look back toward speaker 2. The correlation between the two measures was significant (Pearson’s correlation, *t* (48) = -4.064, *p* < 0.001): cats showing greater surprise were quicker to look back at speaker 2. In Exp.2 we examined whether similar socio-spatial cognitive processing would apply with conspecific rather than human auditory stimuli.

## Experiment 2

Would cats mentally map the location of a familiar cat from its vocalization? To study this, we recorded cats’ “meow” when they were alone and used these calls as stimuli. We chose “meow” because many cats often emitted this vocalization when they were alone. If cats do map the location of a familiar cat, then they might react with surprise when they suddenly hear the latter’s vocalization coming from a different location.

### Methods

#### Subjects

Forty-five cats (22 males, 23 females) participated. Sixteen cats were also tested in Exp.1 (see details in S2 Table), which was conducted at least 6 months earlier. All cats (mean age 3.96 years, *SD* = 2.80 years, range 5 months to 9.75 years) were housed in four different “cat cafés” (mean number of cats living together: 17.25, *SD* = 10.25, range 7 to 30).

#### Apparatus & stimuli

To record “meows” from individual cats, an experimenter placed a sound recorder (Tascam DR-05, USA) with a Sennheiser ME66+K6 super-cardioid shotgun condenser microphone on the floor of the room and left the cat alone in the room for between 30 sec and 5 min. Recording in lossless WAV format (44.1 kHz; 24bit), we thus obtained 6 to 11 “meow” isolation calls, emitted by kittens and adult cats when they are separated from their mother or caretaker, respectively [32, 33]. Vocalizations were obtained from three to five cats per café (for details see S4 Table). The vocalizations were edited to 1 sec in duration (average = 0.923 sec, *SD* = 0.30) and adjusted to the same volume using version 2.3.0 of Audacity® described in the General procedure section [26]. Other settings and apparatus were the same as in Exp.1. Note that we used only familiar cats’ vocalizations to avoid any potential excessively strong reactions to vocalizations from an unfamiliar cat.

#### Analysis

Data from trials with sound error (6 trials) or camera error (1) were excluded from the analysis. One cat was tested in just one condition because she slept on high furniture, out of reach after the first trial. In total, 82 clips were created the same way as in Exp.1; 21 clips for Same_sound_Same_location_, 21 clips for Diff_sound_Same_location_, 21 clips for Same_sound_Diff_location_, and 19 clips for Diff_sound_Diff_location_.

We conducted the same statistical analysis as in Exp.1. Eight blind raters (four men, four women, mean age 23.5 years) scored the degree of surprise to test stimuli masked with pure tones, in a random order for each cat. For latency to look at speaker 2, data were analyzed for 12, 13, 10 and 7 cats who looked back during test phase for Same_sound_Diff_locaation_, Diff_sound_Diff_location_, Diff_sound_Same_location_ and Same_sound_Same_location_ conditions, respectively. We ran a GLM test in which sound (same/diff), location (same/diff) and interaction were entered as fixed factors with Gamma distribution as in Exp.1. We computed the correlation coefficient between magnitude of surprise and latency to look back at speaker 2, using Pearson’s product-moment correlation test.

### Results & discussion

#### Magnitude of surprise

**Fig 4:**
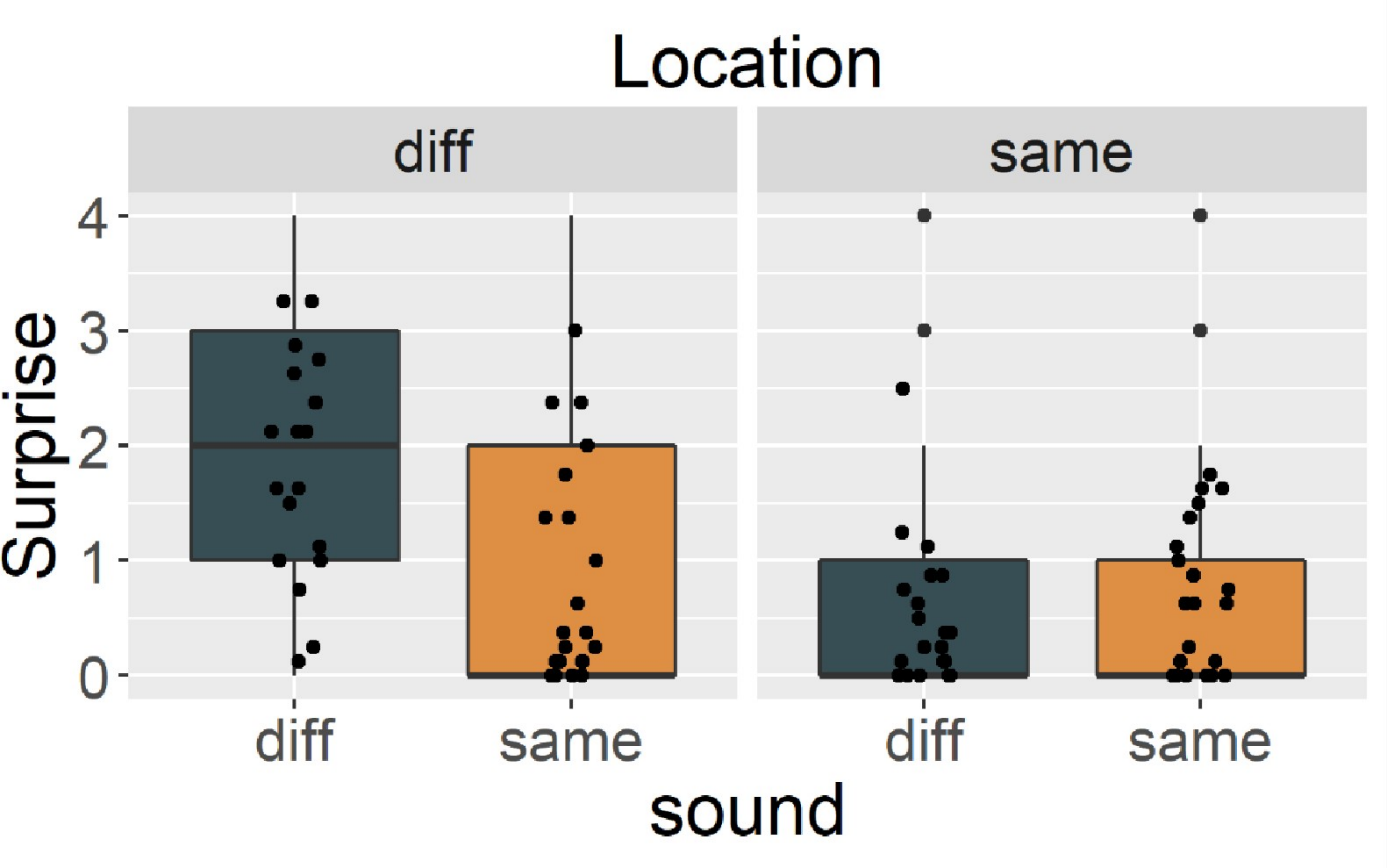
The magnitude of subjects’ surprise rated by eight blind raters in Exp.2. Black blue bar represents Diff_sound_. Orange bar represents Same_sound_. Left panel represents Diff_location_ and right panel represents Same_location_. Each point represents mean magnitude of surprise for each trial.

Fig 4 shows the magnitude of surprise responses. Cats appeared most surprised in the Diff_sound_Diff_location_ condition when another cat’s meow was played back in a different location than the first stimulus; this result did not fit our prediction. LMM revealed significant main effects of location (*X*^2^ (1) = 84.231, *p* < .001) and sound (*X*^2^ (1) = 23.160, *p* < .001), trial order (*X*^2^ (1) = 5.814, *p* = .015), and interactions between location and sound (*X*^2^ (1) = 58.980, *p* < .001). Multiple comparisons showed significant differences between Same_sound_Diff_location_ and Diff_sound_Diff_location_ (*t*(644) = 8.866, *p* < .001) and between Diff_sound_Diff_location_ and Diff_sound_Same_location_ (*t*(644) = 11.909, *p* < .001).

To explain these results, two points should be considered. First, the isolation calls were probably more emotional in content than the voices calling subjects’ names in Exp.1, eliciting a response that could override any response toward the impossible teleportation. Second, adult cats do not direct “meow” to conspecifics [32]; they only use it when requesting something from a human ([34] as cited in [35]). It is possible that they did not in fact recognize the cat they heard meowing.

#### Latency to look at speaker 2

Fig 3(B) shows the latency to look at speaker 2. Cats did not look differentially in the various conditions. GLM revealed no significant main effects of location (*X*^2^ (1) = 3.317, *p* = .068) or sound (*X*^2^ (1) = 0.003, *p* = .950), and no interaction (*X*^2^ (1) = 0.559, *p* = .454). Again, cats probably looked involuntarily at speaker 2 regardless of the sound it played, as it was a novel stimulus.

#### Correlation between response magnitude and latency

Fig 6(B) shows the relationship between the magnitude of surprise responses as rated by humans and latency to look back toward speaker 2. The correlation was significant (Pearson’s correlation, *t* (40) = -2.473, *p* = 0.017): unlike in Exp. 1, cats reacting with greater surprise took longer to look back at speaker 2. In Exp.3 we used non-social sounds as a control to further assess cats’ response in this expectancy violation situation.

## Experiment 3

To clarify the meaning of the results of Exps.1 and 2, in Exp.3 we used non-social sounds as stimuli. We predicted that cats would not show a strong reaction even in the Same_sound_Diff_location_ condition, because this would not represent an “impossible teleportation”.

### Methods

#### Subjects

Forty-seven domestic cats (24 males, 23 females) participated. Sixteen cats were tested in at least one of the previous experiments (see details in S3 Table). The interval between Exps. 2 and 3 was at least 6 months. Twenty-four cats lived at five “cat cafés” (11 males, 13 females, mean age 2.57 years, *SD* = 1.92 years, range 8 months to 7.4 years), and 23 were house cats (13 males, 10 females, mean age 5.97 years, *SD* = 5.03 years, range 6 months to 18.2 years). An additional seven cats were excluded due to camera error (2 cats), external noise disturbance (2) and bad Bluetooth connection (3).

#### Apparatus & stimuli

We used electronic sounds instead of vocalizations to assess the cats’ behavior when the sounds were played back as in the first two experiments. Ideally, it would be better to use familiar sounds such as a door opening sound to match familiarity with the stimuli used in Exp.1. However, it was difficult to equalize the intensity of the stimuli for each house or cat café. We used six electronic sounds downloaded from a web site with many available sounds. S5 Table shows the details of the sounds. All sound stimuli were around 1 sec. (average duration = 1.01 sec., *SD* = 0.01), with volume adjusted using Audacity® [26]. Depending on condition, one or two sound stimuli were randomly chosen for each cat.

#### Analysis

Data from trials spoiled by camera error (2 trials) and sound error (8 trials) were excluded from the analysis. One subject was tested in just one condition to equate the number of subjects in each condition. In total, 83 clips were created: 22 Same_sound_Same_location_, 21 Diff_sound_Same_location_, 20 Same_sound_Diff_location_, and 21 Diff_sound_Diff_location_.

We conducted rating analyses as in Exps. 1 and 2. Another eight blind raters (four men, four women, mean age = 26.5 years) scored the subjects’ degree of surprise to the now-masked test stimuli. The raters observed the 83 clips in random order. For latency to look at speaker 2, data from 12, 13, 9 and 8 cats who looked back toward speaker 2 were analyzed during the test phase for Same_sound_Diff_locaation_, Diff_sound_Diff_location_, Diff_sound_Same_location_ and Same_sound_Same_location_ conditions, respectively. We ran GLM test in which sound (same/diff), location (same/diff) and interaction were entered as fixed factors with Gamma distribution as in the previous experiments. The correlation between magnitude of surprise and latency to look back at speaker 2 was computed as in the two previous experiments.

### Results & discussion

#### Magnitude of surprise

**Fig 5:**
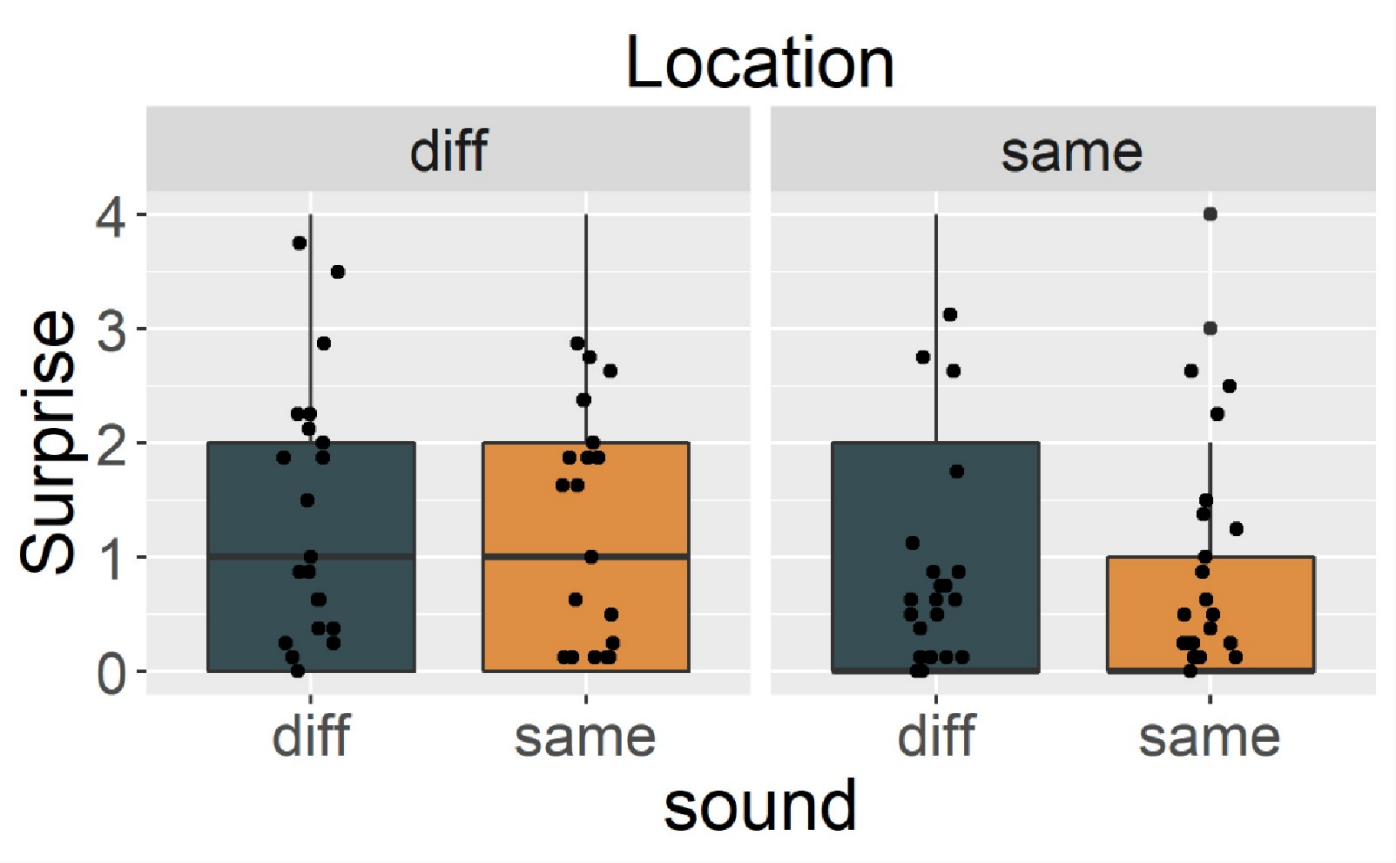
The magnitude of subjects’ surprise rated by eight blind raters in Exp.3. Black blue bar represents Diff_sound_. Orange bar represents Same_sound_. Left panel represents Diff_location_ and right panel represents Same_location_. Each point represents mean magnitude of surprise for each trial.

Fig 5 shows the magnitude of surprise reactions. Cats reacted more when a non-social sound was played from a different location than the first (habituation) sound, regardless of whether or not it was the same sound. LMM revealed significant main effects of location (*X*^2^ (1) = 36.654, *p* < .001) and trial order (*X*^2^ (1) = 20.667, *p* < .001). There was no significant main effect of sound (*X*^2^ (1) = 0.881, *p* = .347) nor an interaction between location and sound (*X*^2^ (1) = .045, *p* = .830). These results suggest that cats simply reacted to a sound from an unexpected location; they did not react differently when the same sound was played from a different location, which contrasts sharply with reaction observed in Exp.1.

#### Latency to look at speaker 2

Fig 3(C) shows the latency to look toward speaker 2. Cats looked back at speaker 2 in the Diff_location_ conditions. GLM test revealed a significant main effect of location (*X*^2^ (1) = 6.048, *p* = .013), but not sound (*X*^2^ (1) = 0.003, *p* = .950), and no interaction (*X*^2^ (1) = 3.155, *p* = .075). Their looks toward speaker 2 probably simply reflect high sensitivity to sounds from unpredicted locations.

#### Correlation between response magnitude and latency

**Fig 6:**
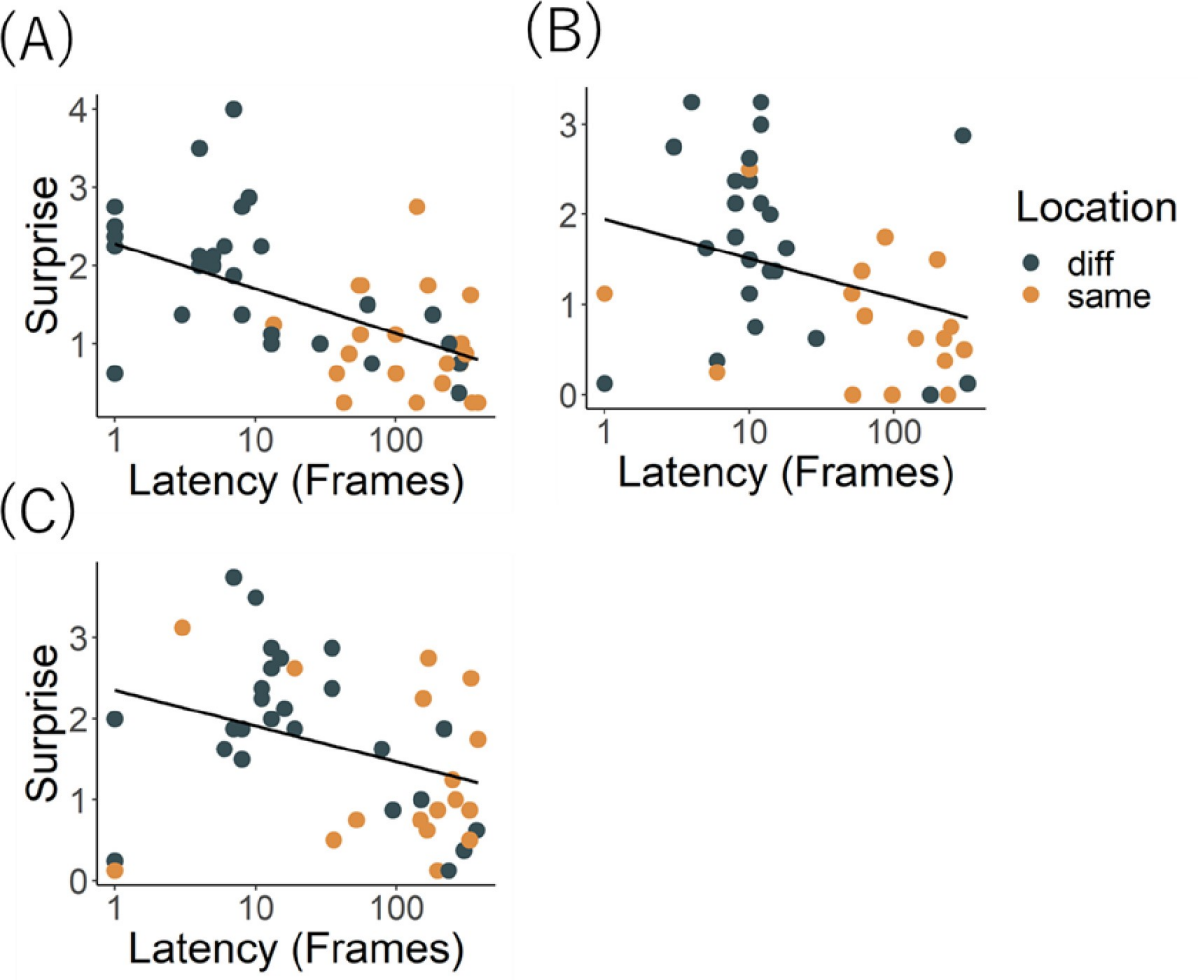
Correlation between latency to look back at speaker 2 and the magnitude of “surprise” as rated in (A) Exp.1, (B) Exp.2 and (C) Exp.3. Orange point represents Same_location_, blue black points represent Diff_location,_ regardless of Sound condition.

Fig 6(C) shows the relationship between magnitude of the surprise response as rated by humans and latency to look back toward speaker 2. The correlation was significant (Pearson’s correlation, *t* (40) = -3.051, *p* = 0.004): similar to Exp. 1, cats showing greater surprise were quicker to look back at speaker 2.

## General discussion

We asked whether cats mentally map other individuals’ locations from their voices. Cats were tested with the owner’s voice (Exp. 1), a familiar cat’s vocalization (Exp. 2) and non-social sounds (Exp. 3), with the habituation and test stimuli played sequentially from two spatially separated speakers. We predicted that cats would be more surprised when the first individual heard was apparently teleported, based on the auditory stimuli, in Exps. 1 and 2. In contrast, we predicted that no such response would occur when the sounds were non-social (Exp. 3). Results showed that cats were surprised when their owner appeared to be “teleported” to a new, unexpected location, but they did not react in the same way when tested with non-social stimuli. These results suggest that cats hold a mental representation of the unseen owner and map their owner’s location from the owner’s voice, showing evidence of socio-spatial cognition.

As stated in the Introduction, socio-spatial cognition appears strongly related to object permanence, the facility which allows children to eventually hold a representation of an object that goes out of sight [1]. Cats succeed in “visible displacement” tasks where they maintain a representation of an object that was previously in sight but no longer is [14]. We found that cats show a similar ability using only auditory information; they maintain a representation of their unseen owner from her voice. We cannot conclude that cats understand “invisible displacement” from our results because it is unclear whether cats were surprised by owner’s presence in an unexpected location or by her apparent absence in the expected location. To address this issue, it would be useful to add a “plausible” condition with enough time between the final habituation sound and the test sound for the stimulus individual to have moved from the first to the second location, as in a study with vervet monkeys [1].

In Exp.2, cats showed no surprise when a familiar conspecific was apparently teleported, unlike in vervet monkeys and meerkats [1, 16]. In our experiment, cats reacted more in the Diff_sound_Diff_location_ condition than any other condition. In contrast to vervet monkeys and meerkats, in which the vocal communication repertoire develops through living in a social group [36, 37], the ancestral relative of the modern domestic cat, the Libyan wildcat (*Felis Lybica*), is a solitary species. Vocal communication among adult cats is limited to two situations, namely fighting and sexual intercourse, although it is more prevalent in kitten-mother and human-cat interactions [32]. For example, Noë and Laporte used vervet monkey affiliative “contact grunts” as stimuli, because they believed that these vocalizations were ecologically valid for testing the monkeys’ socio-spatial cognition [1].

The cat vocalizations used in Exp.2 might be unsuitable for examining socio-spatial cognition for at least four reasons. First is the emotional valence of the “isolation call,” which might have masked any reaction to the “impossible teleportation” event. Second, perhaps cats do not individually recognize conspecifics only from their vocalizations, although they appear to discriminate their owner’s voice from a stranger’s voice [23] and have a cross-modal concept of their owner [22]. Third, given the difference in auditory range between humans and cats (20 Hz to 20 kHz, and 55 Hz to 79 kHz respectively; see [20, 38]), information critical for individual recognition might not have been emitted by the speakers we used. Although we know that cats discriminate conspecific emotional vocalizations (negative vocalization: “Hiss”, positive vocalization: “Purr”) [24] from speakers designed for human hearing, it remains unknown whether they discriminate “meow” vocalizations and individual identity from stimuli played from such speakers. Fourth, “meow” was possibly inappropriate as a stimulus, as adult cats direct this vocalization only to humans, not conspecifics ([34] as cited in [35]).

Might different degrees of familiarity of the sounds have affected the data in Exps.1 and 3, given that we used familiar (owner) vs unfamiliar (stranger) voices in Exp.1 and unfamiliar vs unfamiliar sounds in Exp.3. Cats show caution in the presence of strangers [39] and they discriminate owner from stranger only from voices [23], therefore we predicted surprise reaction from cats upon hearing a stranger’s voice from an unexpected location. However, the magnitude of responses in Exp. 1 was higher when the habituated owner’s voice came from a different location than when a stranger’s voice came from the different location. This finding supports our conclusion that cats mentally mapped their owner’s location from her voice. Ideally, future work will include one familiar person’s voice as the habituation stimulus and another familiar person’s voice as the test stimulus, to further clarify the role of familiarity with the humans in this task.

In Exp.3, we used electronic sounds as auditory stimuli instead of an object sound tied to a specific object (e.g., the squeak of a dog toy is tied to the toy). Some argued that these sounds would not be appropriate as control stimuli because we used voices tied to specific person in Exp.1. Further studies should use the sound tied to some object to confirm that cats do not strongly react to the condition where habituated sounds are played back from a novel location as seen in Exp.1.

In Exps.2 and 3 we found a trial order effect: cats reacted more in the first trial than second trial regardless of the conditions. Interestingly, we did not find this effect in Exp.1. As a trial order effect probably reflects response to stimulus strength or simply novelty rather than the experimental condition, the absence of such an effect actually strengthens our interpretation: cats probably reacted to the “impossible movement of owner” in Exp.1, but to mere novelty of location or sound in Exps.2 and 3.

Our main results are based on magnitude of cats’ responses as judged by human raters. Although the latter were not animal behavior experts, previous studies have indicated that familiarity with animal subjects is not critical for rating many of behaviors. Tami and Gallagher demonstrated that different types of observers including dog-owners, veterinarians, dog trainers and non-owners classified dogs’ behaviors (e.g., as friendly or aggressive) correctly [40], regardless of degree of familiarity with dogs (see also [23, 41]). Furthermore, agreements between raters in our three experiments, as indicated by Cronbach’s coefficient alphas, were high (Exp.1: 0.93, Exp.2: 0.96, Exp.3: 0.97).

We found no clear overall differences in latency, although there were significant correlations between the magnitude of surprise reactions and cats’ latency to look back at speaker 2. It seems likely that wide variability among cats reduced the likelihood of finding overall differences among conditions. Some cats stared toward the owner’s “original” location, whereas other cats moved around the room when the test stimulus was presented. Previous studies have discussed dissociation between rating and coding outcomes and proposed that “subjective” ratings are superior when the targeted concept consists of multiple behaviors. Kubinyi, Gosling, and Miklósi investigated personality in dogs and found that video rating of activity-impulsivity correlated with the scale scores of the owner, but video coding did not [42] (see also [43]). In this study, raters rated cats’ surprise responses expressed by a combination of behaviors including moving ears, head direction, looking back and displace, not just latency to look back; we would argue that this approach is valid for detecting surprise.

Our finding that cats mentally map their owner’s location from their voice corresponds at least to visible displacement in object permanence. Further studies on invisible displacement could benefit from using auditory stimuli, as indeed could similar research on other species. Mentally representing the outside world and manipulating those representations flexibly is an important feature in complex thinking and a fundamental aspect of cognition. Continuing comparative research on this ability can help shed light on how intelligence has evolved in various species and the potential influence of ecological factors.

## Supporting information

S1 Table Subject information in Exp.1. Subjects’ age, sex, living, whether they join the other experiment, error type of trial if a trial was excluded in Exp.1.
(DOCX)

S2 Table Subject information in Exp.2. Subjects’ age, sex, which cat café, whether they join the other experiment, error type of trial if a trial was excluded in Exp.2.
(DOCX)

S3 Table Subject information in Exp.3. Subjects’ age, sex, living, whether they join the other experiment, error type of trial if a trial was excluded in Exp.3.
(DOCX)

S4 Table Details for cats’ vocalizations used in the Exp.2. (DOCX)

S5 Table Details for physical sounds used in the Exp.3. (DOCX)

S1 Video Cats’ typical reaction rated by “4”. (WMV)

S2 Video Cats’ typical reaction rated by “0”. (WMV)

S1 Data Dataset of Exp.1. (CSV)

S2 Data Dataset of Exp.2. (CSV)

S3 Data Dataset of Exp.3. (CSV)

S4 Data Latency data. (CSV)
