# Supplementary material for: Socio-spatial cognition in cats: Mentally mapping owner’s location from voice
Source: PLoS One. 2021 Nov 10;16(11):e0257611. doi: 10.1371/journal.pone.0257611 (PMC8580247; doi:10.1371/journal.pone.0257611)
Supplement: S1 Table — Subjects’ age, sex, living, whether they join the other experiment, error type of trial if a trial was excluded in Exp.1. (DOCX) [file pone.0257611.s001.docx]

| N  S1 Table | age(month) | sex | living | **Join Expeirment** | Error type |
| --- | --- | --- | --- | --- | --- |
| N1 | 48 | F | House | **1** | second trial camera error |
| N2 | 79 | M | Cat café | **1,2,3** |  |
| N3 | 30 | F | Cat café | **1,2,3** | first trial sound error |
| N4 | 72 | F | House | **1** |  |
| N5 | 80 | M | House | **1** |  |
| N6 | 62 | M | House | **1** |  |
| N7 | 20 | M | House | **1** |  |
| N8 | 75 | F | House | **1** |  |
| N9 | 48 | M | House | **1** | first trial camera error |
| N10 | 171 | F | House | **1** | second trial sound error |
| N11 | 46 | M | House | **1** |  |
| N12 | 67 | F | House | **1** |  |
| N13 | 96 | M | House | **1** | first trial camera error |
| N14 | 188 | F | House | **1** |  |
| N15 | 35 | F | House | **1** | first trial camera error |
| N16 | 36 | M | Cat café | **1,2,3** |  |
| N17 | 13 | F | House | **1** |  |
| N18 | 18 | M | Cat café | **1** |  |
| N19 | 18 | M | Cat café | **1,2,3** | second trial camera error |
| N20 | 35 | F | Cat café | **1** | second trial experimental error |
| N21 | 11 | M | House | **1** |  |
| N22 | 147 | M | Cat café | **1** |  |
| N23 | 88 | F | House | **1** |  |
| N24 | 141 | M | House | **1** | first trial sound error |
| N25 | 116 | M | House | **1** |  |
| N26 | 18 | F | House | **1** |  |
| N27 | 57 | M | House | **1** | second trial camera error |
| N28 | 147 | F | Cat café | **1,2** |  |
| N29 | 72 | F | Cat café | **1** | first trial sound error |
| N30 | 86 | F | Cat café | **1,2** | first trial sound error |
| N31 | 47 | M | Cat café | **1,2,3** | second trial sound error |
| N32 | 48 | M | Cat café | **1,2,3** |  |
| N33 | 55 | F | Cat café | **1,2** |  |
| N34 | 34 | F | Cat café | **1,2,3** |  |
| N35 | 98 | M | House | **1** |  |
| N36 | 82 | F | House | **1** |  |
| N37 | 16 | M | Cat café | **1,2,3** | first trial sound error |
| N38 | 48 | F | Cat café | **1,3** | first trial experimental error |
| N39 | 31 | F | Cat café | **1,2** | first trial sound error |
| N40 | 77 | F | Cat café | **1,3** |  |
| N41 | 20 | F | Cat café | **1** |  |
| N42 | 73 | F | Cat café | **1,2** |  |
| N43 | 61 | F | Cat café | **1,3** |  |
| N44 | 146 | F | Cat café | **1** |  |
| N45 | 27 | F | Cat café | **1,3** |  |
| N46 | 102 | F | Cat café | **1** |  |
| N47 | 29 | M | Cat café | **1** |  |
| N48 | 28 | M | Cat café | **1,2** | first trial camera error |
| N49 | 76 | M | House | **1** |  |
| N50 | 11 | M | Cat café | **1** | second trial sound error |
