# Supplementary material for: Socio-spatial cognition in cats: Mentally mapping owner’s location from voice
Source: PLoS One. 2021 Nov 10;16(11):e0257611. doi: 10.1371/journal.pone.0257611 (PMC8580247; doi:10.1371/journal.pone.0257611)
Supplement: S2 Table — Subjects’ age, sex, which cat café, whether they join the other experiment, error type of trial if a trial was excluded in Exp.2. (DOCX) [file pone.0257611.s002.docx]

S2 Table.

| N | Age (month) | sex | Cat café | Join Expeirment | Error type |
| --- | --- | --- | --- | --- | --- |
| v1 | 46 | F | A | 1,2,3 |  |
| v2 | 38 | M | A | 1,2 | first trial camera error |
| v3 | 12 | M | A | 2 |  |
| v4 | 61 | F | C | 1,2 |  |
| v5 | 61 | M | C | 1,2,3 |  |
| v6 | 43 | F | C | 1,2 | second trial sound error |
| v7 | 109 | F | C | 1,2 |  |
| v8 | 14 | M | D | 2 |  |
| v9 | 17 | M | D | 2,3 |  |
| v10 | 20 | M | D | 2,3 |  |
| v11 | 22 | M | D | 2,3 | first trial sound error |
| v12 | 30 | M | C | 1,2,3 |  |
| v13 | 48 | F | C | 1,2,3 |  |
| v14 | 53 | M | C | 1,2 |  |
| v15 | 35 | M | A | 1,2,3 | second trial sound error |
| v16 | 85 | M | A | 1,2,3 | second trial sound error |
| v17 | 63 | M | C | 1,2,3 |  |
| v18 | 71 | F | C | 1,2 | first trial sound error |
| v19 | 85 | F | C | 2 | first trial sound error |
| v20 | 69 | F | D | 2 |  |
| v21 | 117 | F | B | 2 |  |
| v22 | 117 | M | B | 2 |  |
| v23 | 96 | M | B | 2 |  |
| v24 | 42 | M | D | 2 |  |
| v25 | 18 | F | D | 2,3 |  |
| v26 | 5 | M | D | 2 |  |
| v27 | 5 | F | D | 2 |  |
| v28 | 16 | F | C | 2,3 |  |
| v29 | 45 | F | C | 1,2 |  |
| v30 | 43 | M | C | 2 |  |
| v31 | 6 | F | D | 2 |  |
| v32 | 6 | M | D | 2 |  |
| v33 | 31 | F | D | 2 |  |
| v34 | 21 | F | C | 2,3 |  |
| v35 | 18 | F | C | 2 |  |
| v36 | 80 | F | C | 1,2 |  |
| v37 | 8 | F | C | 2 |  |
| v38 | 44 | M | C | 2 |  |
| v39 | 47 | M | C | 2 |  |
| v40 | 73 | F | B | 2 |  |
| v41 | 121 | M | B | 2 |  |
| v42 | 11 | F | C | 2 |  |
| v43 | 68 | M | C | 2 |  |
| v44 | 96 | F | C | 1,2 | second trial cat slept in the place out of our reach |
| v45 | 24 | F | D | 2 |  |
