# Supplementary material for: Socio-spatial cognition in cats: Mentally mapping owner’s location from voice
Source: PLoS One. 2021 Nov 10;16(11):e0257611. doi: 10.1371/journal.pone.0257611 (PMC8580247; doi:10.1371/journal.pone.0257611)
Supplement: S3 Table — Subjects’ age, sex, living, whether they join the other experiment, error type of trial if a trial was excluded in Exp.3. (DOCX) [file pone.0257611.s003.docx]

S3 Table.

| N | age(month) | sex | living | Join Expeirment | Error type |
| --- | --- | --- | --- | --- | --- |
| c1 | 37 | F | House | 3 |  |
| c2 | 12 | F | Cat café | 3 |  |
| c3 | 31 | F | Cat café | 1,3 |  |
| c4 | 12 | F | Cat café | 3 |  |
| c5 | 73 | M | Cat café | 1,2,3 |  |
| c6 | 23 | M | Cat café | 1,2,3 | second trial camera error |
| c7 | 27 | M | Cat café | 1,2,3 |  |
| c8 | 120 | F | House | 3 |  |
| c9 | 22 | M | House | 3 |  |
| c10 | 6 | M | House | 3 |  |
| c11 | 38 | F | Cat café | 1,2,3 | second trial camera error |
| c12 | 9 | M | House | 3 |  |
| c13 | 53 | M | House | 3 |  |
| c14 | 58 | M | House | 3 | second trial sound error |
| c15 | 44 | F | House | 3 |  |
| c16 | 32 | M | House | 3 |  |
| c17 | 102 | M | House | 3 |  |
| c18 | 214 | M | House | 3 | first trial sound error |
| c19 | 70 | M | House | 3 |  |
| c20 | 70 | M | House | 3 |  |
| c21 | 24 | M | Cat café | 1,2,3 |  |
| c22 | 1418 | F | Cat café | 3 |  |
| c23 | 25 | M | House | 3 |  |
| c24 | 41 | M | House | 3 | second trial sound error |
| c25 | 92 | F | House | 3 |  |
| c26 | 58 | F | House | 3 |  |
| c27 | 35 | M | House | 3 |  |
| c28 | 178 | F | House | 3 | second trial sound error |
| c29 | 60 | M | Cat café | 1,2,3 |  |
| c30 | 1421 | F | Cat café | 3 | second trial sound error |
| c31 | 10 | F | Cat café | 2,3 |  |
| c32 | 59 | M | Cat café | 1,2,3 |  |
| c33 | 18 | M | Cat café | 2,3 |  |
| c34 | 12 | F | Cat café | 2,3 |  |
| c35 | 20 | M | Cat café | 2,3 |  |
| c36 | 60 | F | Cat café | 3 |  |
| c37 | 14 | F | Cat café | 3 | first trial sound error |
| c38 | 89 | F | Cat café | 1,3 |  |
| c39 | 46 | F | Cat café | 1,2,3 |  |
| c40 | 51 | M | Cat café | 1,2,3 |  |
| c41 | 9 | F | House | 3 | second trial sound error |
| c42 | 219 | F | House | 3 | first trial sound error |
| c43 | 15 | M | Cat café | 2,3 |  |
| c44 | 81 | F | House | 3 |  |
| c45 | 74 | F | House | 3 |  |
| c46 | 18 | F | Cat café | 3 | second trial not join |
| c47 | 18 | M | Cat café | 3 |  |
