# Supplementary figures and images for: Socio-spatial cognition in cats: Mentally mapping owner’s location from voice
Source: PLoS One. 2021 Nov 10;16(11):e0257611. doi: 10.1371/journal.pone.0257611 (PMC8580247; doi:10.1371/journal.pone.0257611)

S4 Table. Details for cats’ vocalizations used in the Exp.2


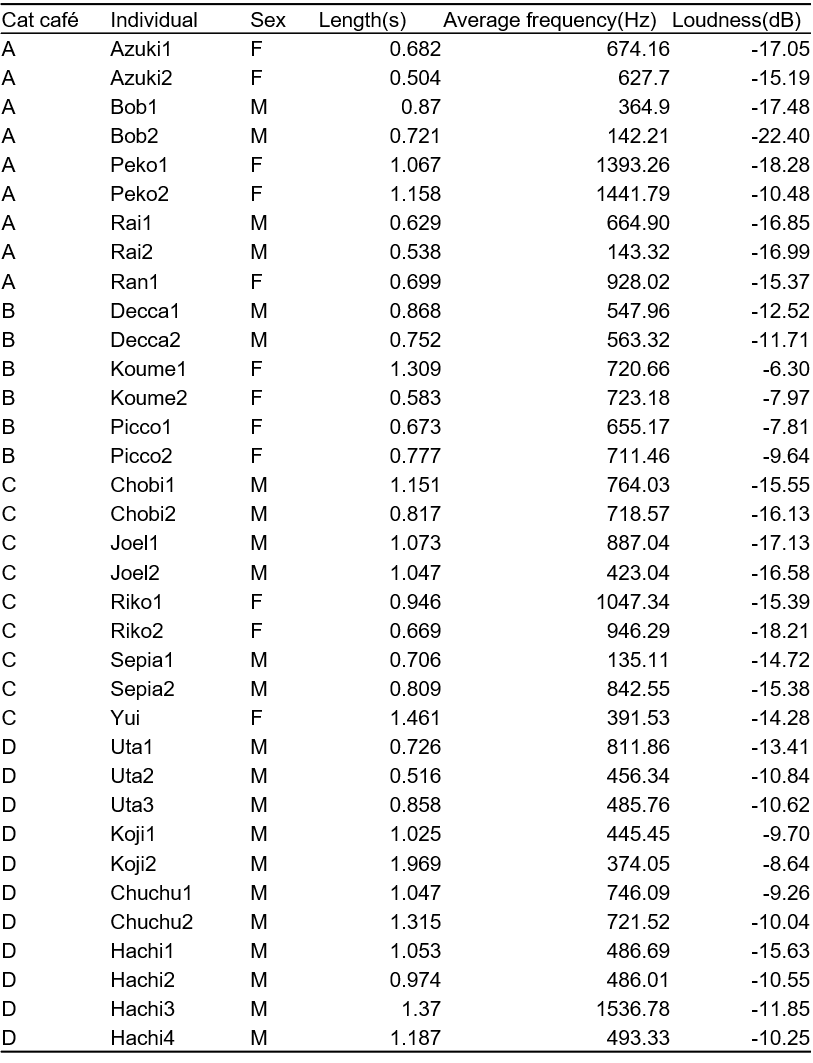

Supplement: S4 Table — (DOCX) [file pone.0257611.s004.docx]

S5 Table. Details for physical sounds used in the Exp.3


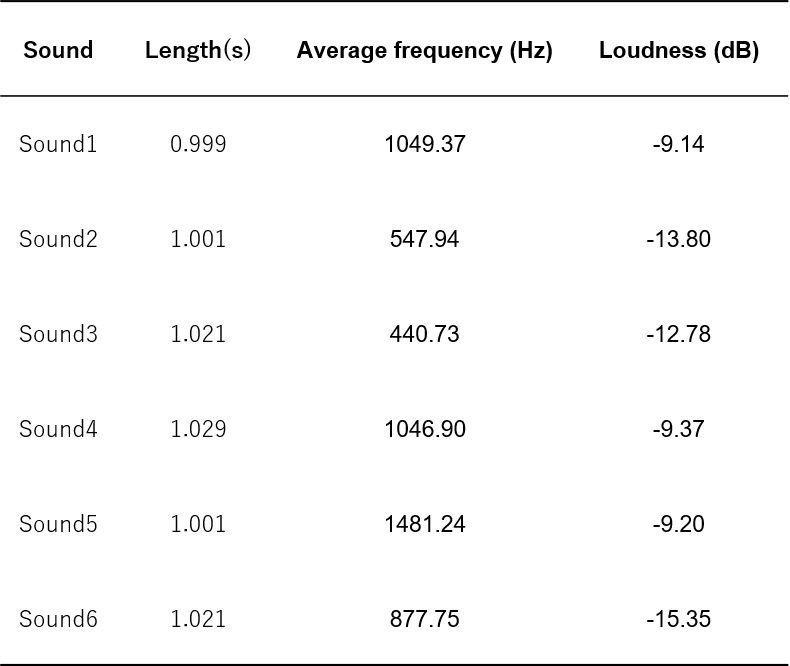

Supplement: S5 Table — (DOCX) [file pone.0257611.s005.docx]
